# Scabies Exacerbation With Eosinophilia As Immune Reconstitution Inflammatory Syndrome in a Patient With AIDS and Pneumocystis Pneumonia: A Case Report

**DOI:** 10.7759/cureus.111689

**Published:** 2026-06-28

**Authors:** Yu Kasamatsu, Masanori Kobayashi, Kyoko Yagyu, Tetsushi Goto, Michinori Shirano

**Affiliations:** 1 Infectious Disease, Osaka City General Hospital, Osaka, JPN; 2 Respiratory Medicine, Osaka City Juso Hospital, Osaka, JPN; 3 Infectious Disease, Osaka City Juso Hospital, Osaka, JPN

**Keywords:** aids, eosinophilia, hiv, immune reconstitution inflammatory syndrome, scabies

## Abstract

Only a few studies have reported the relationship between immune reconstitution inflammatory syndrome (IRIS) and scabies. A 30-year-old homosexual man who lived in a dorm was diagnosed with AIDS based on his CD4 count: 10/μL, HIV-1 real-time PCR: 100000 copies/mL, and pneumocystis pneumonia (PCP). Although he developed a rash on his face and back shortly before, it exacerbated rapidly, and a rise of eosinophils was noted in his blood after initiation of antiretroviral therapy. Initially, he was suspected of having IRIS of PCP with eosinophilia, but he was ultimately diagnosed with scabies, which was thought to be exacerbated by IRIS. He recovered completely from his symptoms and eosinophilia after combination therapy of ivermectin and sulfur formulations.

## Introduction

Scabies is a parasitic infection of the skin caused by mites. It occurs in two distinct forms: crusted (Norwegian) scabies and ordinary scabies. Patients with human immunodeficiency virus (HIV) who have a CD4 count <150 are said to be at risk of developing crusted scabies [1]. Although one case of acquired immunodeficiency syndrome (AIDS) with crusted scabies that was rapidly exacerbated by antiretroviral therapy (ART) has been reported, few studies report on the relationship between immune reconstitution inflammatory syndrome (IRIS) and scabies [2-4]. Herein, we report a case of a patient with AIDS who was clinically exacerbated upon administering ART and was considered to be IRIS.

## Case presentation

A 30-year-old homosexual man who lived in a dorm was arrested by the police on suspicion of illegal drug addiction. During the investigation, he was found to have a fever and was taken to a nearby hospital, where he was diagnosed with severe pneumonia and multiple organ failure. He was treated with ventilator management, continuous hemodiafiltration (CHDF), and various antibiotics. However, he responded poorly to the treatment and tested positive for anti-HIV antibody. Consequently, he was transferred to our hospital.

The laboratory data on admission were as follows: WBC, 2000/μL, CD4, 10/μL, and HIV-1 real-time PCR, 100000 copies/mL. In addition to bacterial pneumonia caused by *Pseudomonas aeruginosa* and *Streptococcus pneumonia*, the patient had developed *Pneumocystis* pneumonia (PCP) as a result of AIDS. He was treated for three weeks with 3 g of meropenem daily and 720 mg/3600 mg of trimethoprim/sulfamethoxazole (ST) daily. Once an improvement in his condition was observed, we switched from ST to 1500 mg of atovaquone daily because he developed fever and elevation of aminotransferase. Apart from this, the clinical course of this condition was good, and the patient was taken off the ventilator on hospital day 25. Next, the patient was administered tenofovir/emtricitabine + darunavir/ritonavir on hospital day 33. Moreover, his tracheostomy tube was removed on hospital day 42.

**Figure 1:**
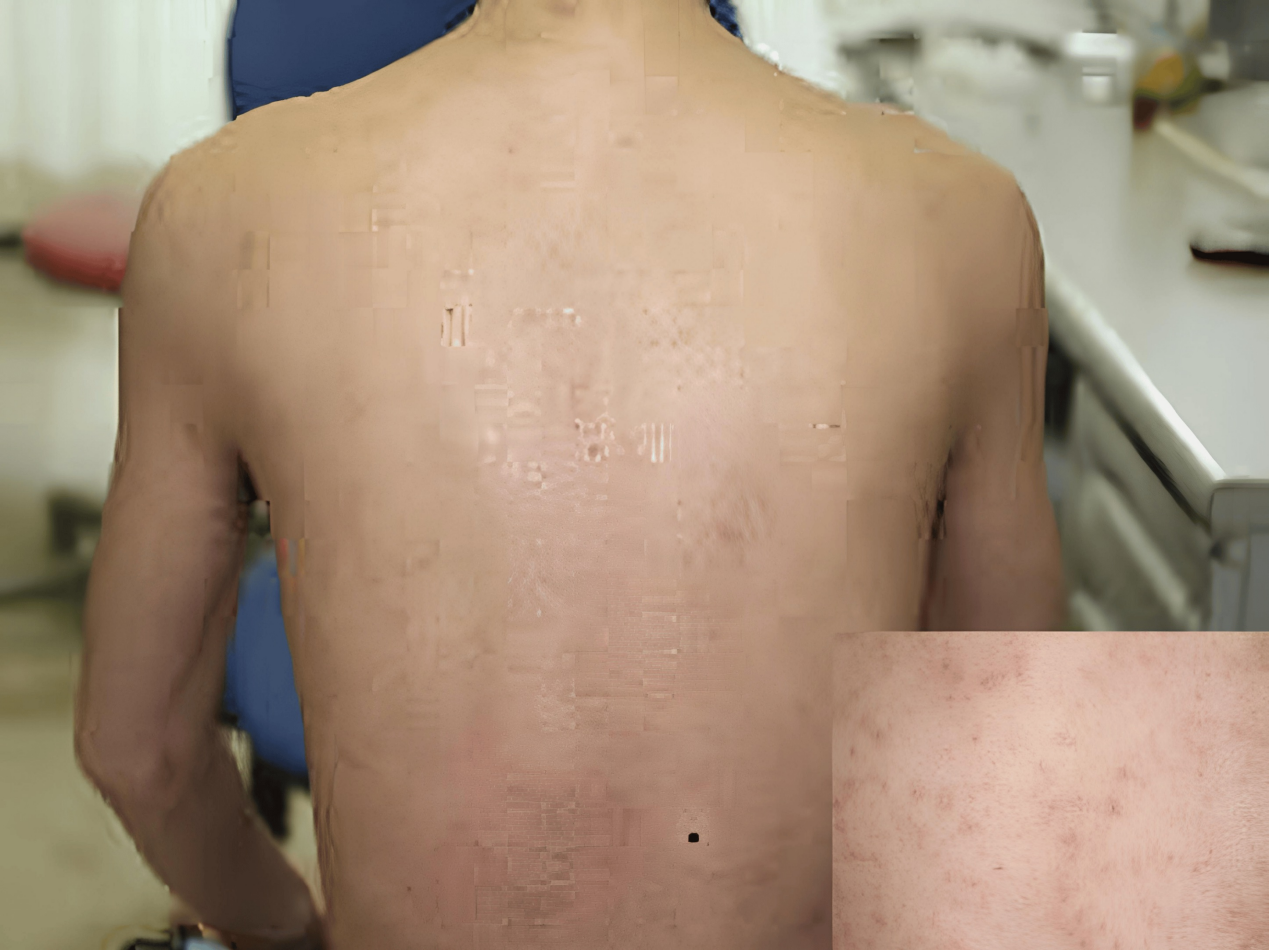
The patient's skin findings Multiple small papules on the back and face.

**Figure 2:**
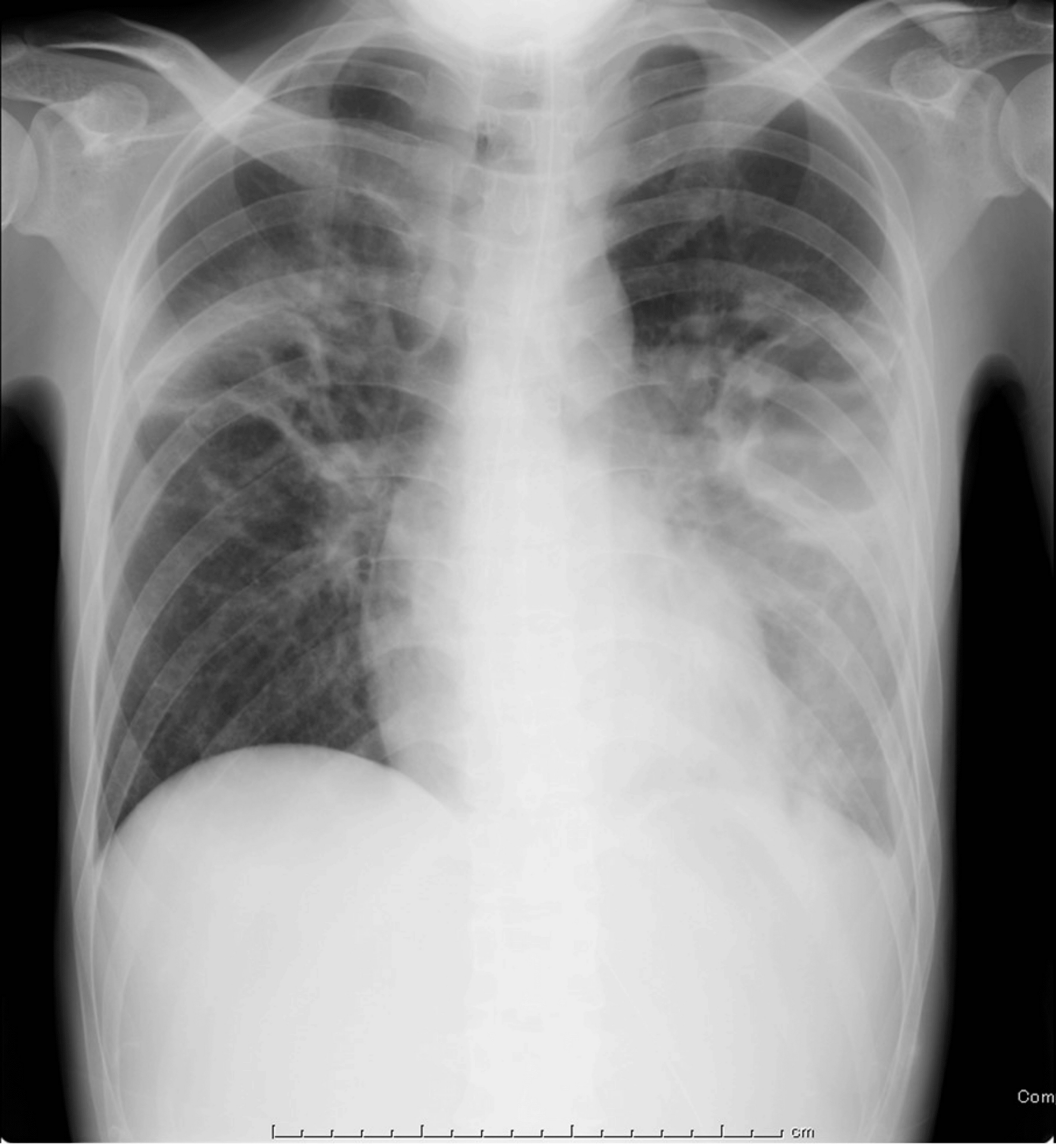
The patient`s chest X-ray The chest X-ray revealed cystic lesions and ground-glass opacities (GGOs) in the left lung field.

Although our patient was undergoing rehabilitation for drug abuse and disuse syndrome, and received topical treatment for pressure ulcer by dermatologists, he developed rashes on his face and back that rapidly worsened from the day after ART was initiated (Figure 1). He complained of a strong itching sensation and developed insomnia. We suspected prolonged antibiotics-induced eczema and seborrheic eczema and administered oral and topical antihistamines. A rapid elevation of eosinophils was seen in his blood, and we suspected IRIS of PCP and conducted various tests. Based on his positive pneumocystis PCR result and abnormal shadows on the chest X-ray (Figure 2), we provisionally diagnosed IRIS of PCP and initiated additional treatment with ST. His fever and respiratory symptoms improved after that, but eosinophilia persisted, and the cause remained unknown at that time.

**Figure 3:**
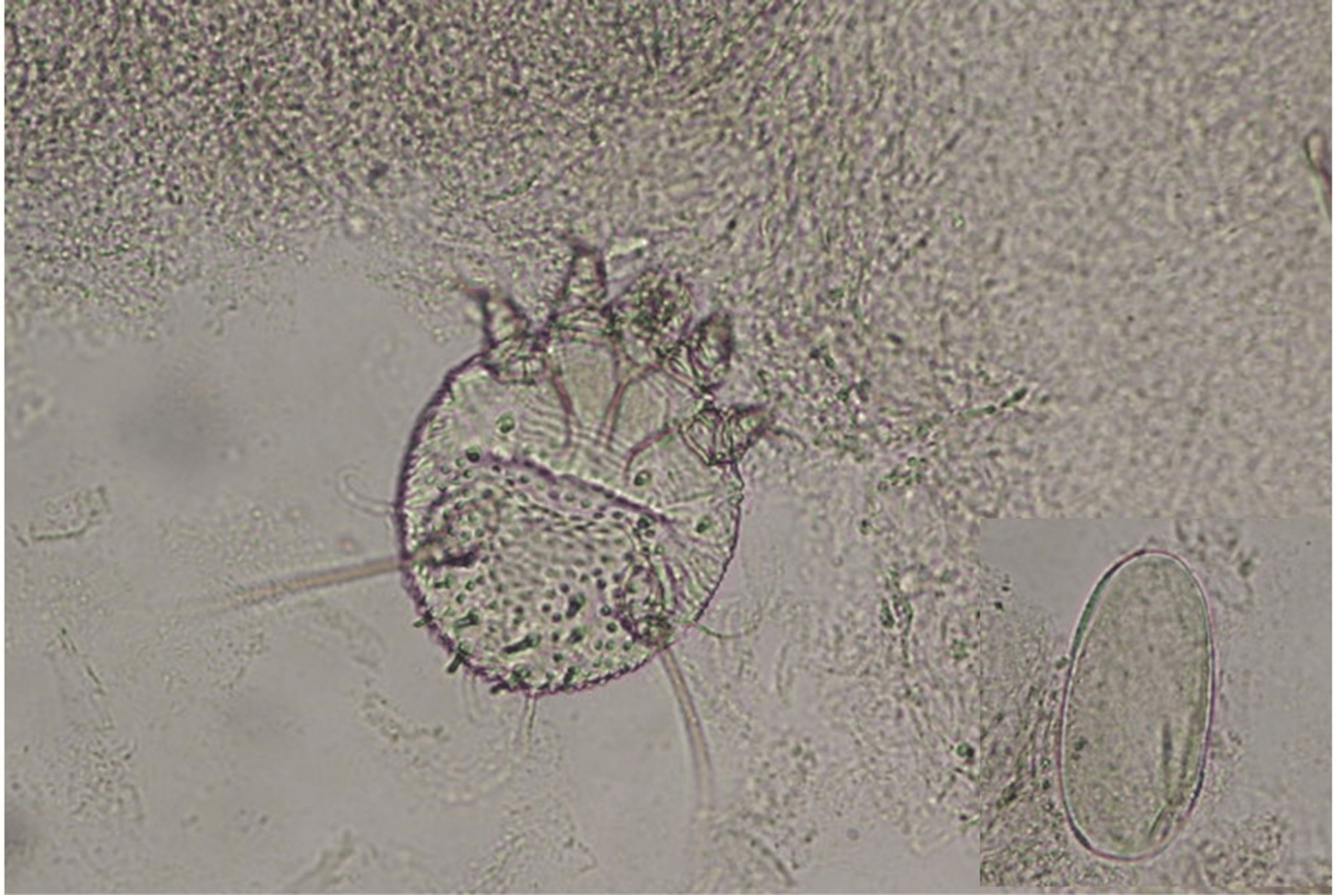
The patient`s microscopic examination Microscopic examination revealed numerous adult scabies mites and eggs (bottom right).

Although the patient was discharged on hospital day 60 because of improvement in his general condition, he revisited the dermatologist for persistent symptoms after discharge. He was diagnosed with scabies upon detection of insect bodies and eggs under microscopic examination (Figure 3). Owing to the migration of the vast number of mites and eggs, progression to crusted scabies was suspected, and the patient was treated with 12 mg of oral ivermectin twice every three weeks and topical treatment using sulfur formulations for six weeks.

**Figure 4:**
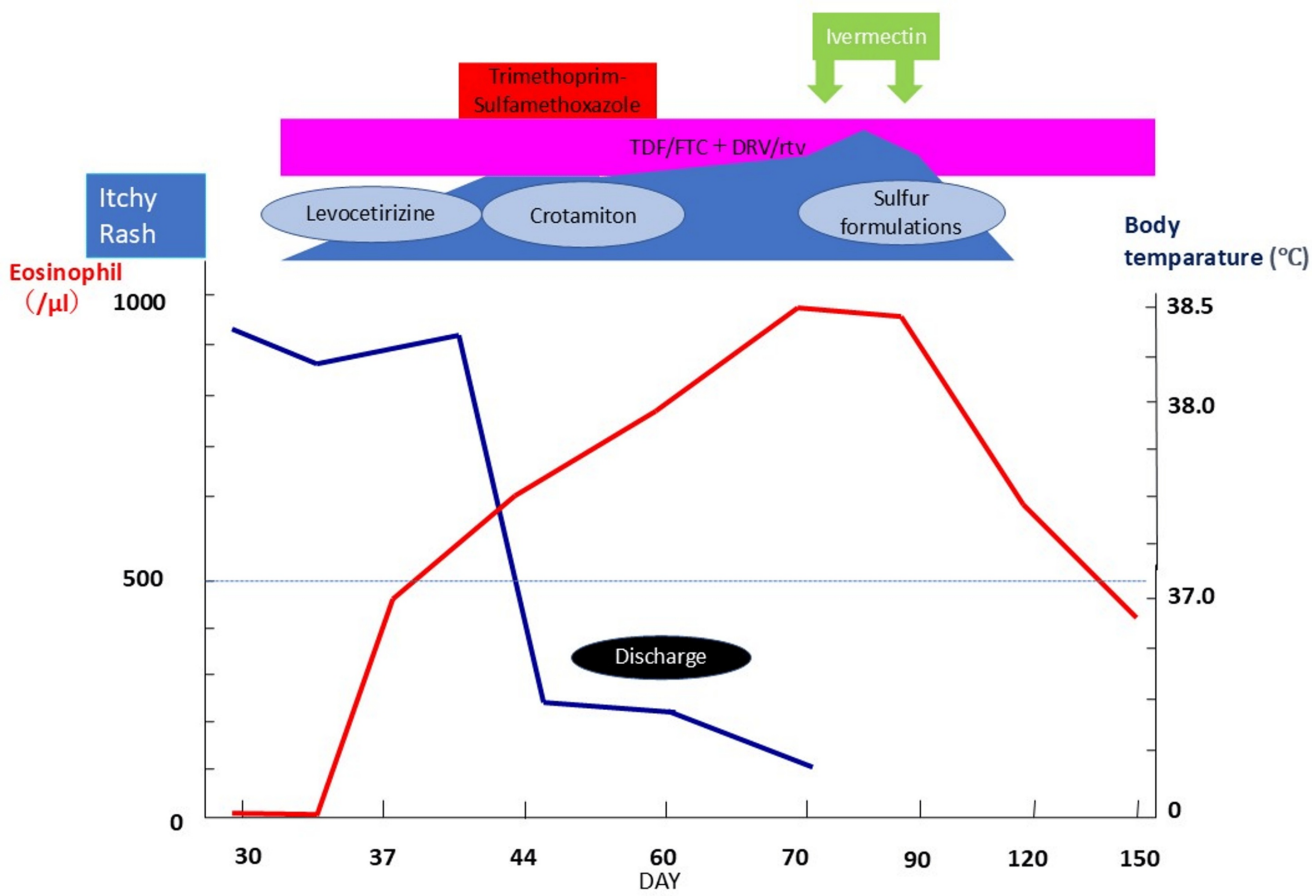
The patient`s clinical course and eosinophil count The patient`s symptoms and eosinophil count improved after combination therapy of ivermectin and sulfur formulations. No artificial intelligence (AI) tool was used to create the image.

At the 1-month follow-up, his symptoms showed a significant improvement, and at the 3-month follow-up, his eosinophil count had normalized (Figure 4).

## Discussion

AIDS-related pruritic diseases are dependent on the immunity of the host and exhibit atypical symptoms, making them difficult to diagnose and treat [1,4]. The ST therapy used for PCP may be mistaken for a side effect, even if the patient complains of itching or other skin symptoms. Scabies is common in persons with HIV, and once the helper T-cell count is less than 200 cells/μL, scabies may present atypically [5,6]. Moreover, one report stated that even if CD4 values are normal, scabies can be misdiagnosed as a fungal infection owing to its atypical manifestation [7]. In our case, the symptoms had been masked by AIDS-related immunosuppression, and they manifested only upon administering ART.

Treatment generally consists of 5% permethrin cream, 10% crotamiton lotion and cream, 1% lindane lotion, ivermectin, and sulfur formulations. We treated our patient with sulfur formulations and ivermectin based on a recommendation mentioned in a previous report on scabies associated with HIV infection [8]. 

The mechanism of scabies in humans is thought to be complicated and is hypothesized to be correlated with the dominance of an IgE-driven Th2 response in severe disease or an interferon-gamma-dominated Th1 response that promotes parasite control [9]. Increases in eosinophil and IgE counts are generally observed in blood tests, and these normalize after treatment [10,11]. Th1 and Th2 cells of CD4-positive T cells decrease remarkably if a patient contracts AIDS, and this decrease causes a reduction in interleukin (IL)-4, IL-5, and granulocyte-macrophage colony-stimulating factor (GM-CSF) levels, all of which are important for parasite immunity [12].

Fernández-Sánchez et al. reported a case in which the symptoms of scabies were initially masked, but rapidly exacerbated to crusted scabies during the course of IRIS after administering ART [2]. Similar cases have also been reported in patients without HIV [3]. Before initiating ART, our patient had complained of itching a little, and his eosinophil count was low. We presume that in our patient, host immunity did not function properly against scabies as in previous cases because of the remarkably low CD4 count of 10/μL at the first visit, despite systemic infection due to numerous insects. We suspect that our patient also developed IRIS because he complained of itching and developed a rash on his entire body because of ordinary scabies and a rise of eosinophils in his blood that had exacerbated rapidly after ART. Although eosinophilia has been reported to occur in conjunction with PCP, sputum PCR testing for *Pneumocystis* in this case ruled out IRIS associated with PCP [13,14].

## Conclusions

We have encountered a case of IRIS in which eosinophil counts rose and eczema and itching developed after the start of ART.　Eczema in patients with HIV is atypical and can have various causes. In patients with AIDS, if eczema and itching worsen during the course of IRIS after initiating ART, physicians should consider the possibility of scabies. In addition, when evaluating elevated eosinophil counts in patients with HIV, it is important to rule out not only parasitic and fungal infections but also scabies. Early detection is important for avoiding a hospital-wide infection.
